# Essential competencies for an effective physician-to-physician teleconsultation

**DOI:** 10.1186/s12909-025-08362-6

**Published:** 2025-11-25

**Authors:** Samane Ghasemi, Tahereh Changiz, Athar Omid

**Affiliations:** Medical Education Research Center, Department of Medical Education, Isfahan University of Medical Sciences, Isfahan, Iran

**Keywords:** Physician, Teleconsultation, Competency, Delphi method

## Abstract

**Introduction:**

The increasing use of teleconsultation deserves attention to highlight the required competencies in planning training and assessment activities. This study aimed to determine the competencies of specialist physicians involved in teleconsultation.

**Methods:**

This cross-sectional descriptive study was conducted through the Delphi method. First, a literature review, interviews, and expert panel discussions were conducted. The areas of competence and competencies required for a specialist physician involved in teleconsultation were extracted. The list of competencies was designed as an electronic questionnaire in two Delphi rounds. Fifty-five specialists involved in teleconsultation were asked to determine the degree of necessity of the competencies. Finally, a second round of Delphi was conducted for the competencies with less than 70% agreement.

**Results:**

Sixty-nine competencies were identified. In the first round of the Delphi, 63 competencies were accepted, for a quota of 70%, and 6 were transferred to the second round of the Delphi. At this round, 3 competencies were removed, and 66 competencies were accepted. The main competency domains were extracted as follows: “receiving and presenting data”, “professional ethics”, “awareness and management of emotions”, “communication skills”, “diagnosis and patient management”, “collaborative decision-making”, and “teleconsultation and use of technology”.

**Conclusion:**

The competencies identified in this study could provide a framework for developing targeted training programs and professional standards in teleconsultation. By integrating these competencies into medical education, healthcare systems can promote the quality and effectiveness of teleconsultations, as such, improving equitable access to health services.

**Supplementary Information:**

The online version contains supplementary material available at 10.1186/s12909-025-08362-6.

## Introduction

Recent years have witnessed significant growth in information and communication technologies (ICTs) in healthcare, enabling teleconsultation and access to medical services [1]. Teleconsultation is defined as synchronous or asynchronous consultation using information and communication technology to eliminate geographical and functional distance [2]; which can be provided through various channels such as telephone, video conferencing systems, instant messaging and mobile applications [3]. There are several types of communication in teleconsultation: both parties are health providers (e.g. physician-to-physician teleconsultation), communication between a physician and a primary care provider such as a nurse, one party is a health provider and the other party is a patient (physician-patient communication), a primary care provider and a patient, and finally a three-way communication between a physician, primary care provider and patient [2].

Physician-to-physician teleconsultation enhances communication and data sharing among healthcare professionals who share responsibilities for patients, such as specialists and general practitioners [4]. It also allows medical professionals to seek advice from peers with specialized knowledge [5]. Research has shown that physician-to-physician teleconsultation in healthcare demonstrates significant benefits, including improved access to specialized care for remote populations, significant cost savings, improved access to specialized care, and enhanced skills of healthcare professionals in managing diverse patients [6, 7]. However, challenges such as technical difficulties, legal issues, and payment concerns persist. Despite these obstacles, many doctors express interest in regularly implementing physician-to-physician teleconsultation services, recognizing their potential to improve healthcare delivery and accessibility, particularly in rural areas and during health crises [8].

The increasing prevalence of teleconsultation in healthcare delivery necessitates proper training for healthcare providers to ensure effective implementation. Studies highlight the importance of integrating teleconsultation education into core medical curricula and continuing medical education [9, 10]. Despite its growing importance, standardized teleconsultation education is often neglected in medical curricula. It seems necessary to incorporate teleconsultation education into the training of healthcare providers to ensure the proper use of this healthcare delivery system [9].

Competency-based training for physician-to-physician teleconsultation is crucial, as it requires the same level of thoroughness and clinical judgment as face-to-face consultations [11]. In Galen et al.‘s study, the key competencies of teleconsultation were effective communication through digital platforms, maintaining patient privacy, and remote management of chronic diseases [12]. Competency-based education (CBE) has become as a focal point of health professions education, aiming to better prepare healthcare professionals for practice worldwide [13]. This approach seeks to educate professionals whose skills align with the needs of the communities they serve and whose clinical abilities meet the demands of healthcare systems [14]. Competency encompasses medical knowledge, skills, attitudes, and metacognitive capabilities, reflecting stakeholder needs and inferred from performance evaluations [15]. Professional training and assessment can be guided by a competency framework [16]. The intended outcome of a CBE-based program is to educate competent health professionals who can practice medicine at a specific level, tailored to local needs [17]. Competency-based education in medical training focuses on defining and assessing specific competencies along a developmental continuum, from novice to expert [18].

Given the rapid advancement of digital technologies in healthcare and the significant increase in physician-to-physician teleconsultations, developing specialized competencies for this practice has become imperative. As physician-to-physician teleconsultation quality directly impacts treatment accuracy and effectiveness, the current lack of structured training programs may compromise service quality and potentially lead to medical errors. To address this gap, this study was designed to answer the research question: “What competencies are essential for effective physician-to-physician teleconsultation?” by identifying and classifying the required competencies.

## Methods

This descriptive and cross-sectional study was conducted in two phases (February 2024 to March 2025). Initially, a literature review and several interviews were conducted, followed by the modified Delphi method in two rounds. The Delphi method is a qualitative technique used to gather expert opinions through iterative rounds of questionnaires and feedback [19]. It is particularly useful when face-to-face interaction is impossible, allowing experts from different geographical locations to participate [15]. As the participants were physicians (either as referrers or consultants) in the teleconsultation process at Iranian universities of medical sciences, this study applied the Delphi method to gather their opinions.

### Literature review and interviews

First, an overview of studies on factors affecting teleconsultation was conducted in eight online databases (Irandoc, Sid, Magiran, ScienceDirect, Medline, WOS, Eric, and Scopus). Then, based on the results extracted from reviewing the studies and consulting with the research team, a few general open-ended questions were formulated, such as “What factors affect the conditions of teleconsulting?”, “What competencies should be considered when training assistants in telecounseling?”, and “How do you manage data collection during teleconsultation?” Participants in the interviews were physicians involved in the teleconsultation at *the Isfahan University of Medical Sciences*. Interviews were conducted in person. Data collection continued until theoretical saturation (*N* = 16). After coding and analyzing the interviews, an expert panel was convened with the participation of several medical education specialists (*N* = 5) and physicians (*N* = 3) involved in teleconsultation, to confirm and categorize the extracted capabilities. As such, the content validity of the Delphi questionnaire was confirmed by the expert panel members. Finally, 69 competencies were extracted, which were categorized based on the role of the physician in the teleconsultation process (referring, consultant, and both roles of referring and consultant). A total of seven domains were selected for competencies, in which a number of competencies were specifically proposed for the referring or the consultant and differed for them, while some were jointly proposed for both.

### First round of Delphi

A 5-point Likert scale electronic questionnaire was developed, with response options including necessary, useful, ineffective, harmful, and I cannot give an opinion. Before completing the survey, participants were briefed on the study’s objectives and provided informed consent. The questionnaire collected demographic data such as age, gender, specialty, years of experience, and prior teleconsultation experience.

After each competency section, participants were asked an open-ended question to suggest additional competencies or provide feedback on those listed. The analysis of responses indicated no new or non-repetitive topics.

In selecting specialists through purposive sampling, we aimed to maximize diversity across medical specialties. 90 specialists who were active in the field of teleconsultation and affiliated with Iranian universities of medical sciences and participated in resident training were invited to participate in the study via email containing a link to the questionnaire and response instructions. The response period was 4 weeks, during which 3 reminders to participate were sent to them. Descriptive statistics (percentages) were used for data analysis, with a predefined consensus threshold of 70% [20] .

### Second Delphi round

Items that did not reach at least 70% consensus were carried over to the second Delphi round. The electronic questionnaire featured six competencies. The same participants from the first round took part in the second round. During this phase, anonymized data from the initial round (including percentage agreement) were shared with the participants alongside the questionnaire, allowing them to reconsider their earlier responses based on this controlled feedback. Following the collection of the second-round responses, the data were analyzed by computing percentages. Competencies that achieved over 70% agreement were retained, while those below this threshold were discarded.

In their study, Niederberger et al. (2024) identified five common characteristics of Delphi studies based on a comprehensive literature review. These characteristics were also evident in the present study and are as follows:

1. Experts were surveyed and their anonymity was maintained.
2. The survey was conducted in two Delphi rounds.
3. A standard questionnaire, often with open-ended questions, was used to collect arguments and draw horizons of legitimacy.
4. The statistical analysis is based on descriptive statistics.
5. From the second Delphi round onwards, experts received feedback on the results of the previous round along with the questionnaire and were therefore able to re-examine their judgments and revise them if necessary [21].

## Results

In the review of studies and interviews conducted by three members of the research team, 122 competencies were extracted, which were discussed in the expert panel. In the expert panel, 69 competencies were classified and approved. These competencies were designed and sent in the form of an electronic questionnaire for the Delphi rounds. The response rate was 61% in both Delphi rounds. Demographic information of participants in the Delphi rounds is presented in Table 1.

**Table 1:** Demographic information

Demographic	Information
Gender	Women: 25 (45.4%) - Men: 30 (54.5%)
Average age	47.6 years, age range: 33–63 years
Average work experience	14.3 years (minimum: 3 years - maximum: 31 years)
Average teleconsultation experience	12.4 years (minimum: 2 years - maximum: 29 years)
Educational fields	- Obstetrics and gynecology (27.2%) - Pediatrics (10.9%) - Plastic and reconstructive surgery (1.8%) - General surgery (5.4%) - ENT (5.4%) - Anesthesia (9.0%) - Urology (9.0%) - Neurology (9.0%) - Orthopedics (12.6%) - Emergency medicine (1.8%) - Internal medicine (5.4%)

Table 2 shows the results of the agreement in the first Delphi round. The highest agreement was 98.1%, and the lowest agreement was 50.9%. In total, out of the 69 competencies endorsed by the experts in the first and second Delphi rounds, 63 competencies achieved an agreement of over 70% in the essential option. Open-ended questions did not add any competencies to Table 2.

**Table 2:** The percentage of agreements in the first round of Delphi

The role of the physician	Competency domains	Competency	necessary	useful	ineffective	harmful	I cannot give an opinion
Referring physician	Receiving and presenting data	1. Classify the patient’s key data and summarize them before contacting the consultant physician.	98.1%	1.8%	0	0	0
		2. Analyze the patient’s data and extract your main question for the consultation.	96.3%	3.6%	0	0	0
		3. Explain the patient’s data, especially the examination and paraclinical data, correctly orally.	92.7%	3.6%	1.8%	0	0
		4. Use specialized language (specialized terminology) to present the data.	81.8%	18.1%	0	0	0
		5. Provide the patient’s data with commitment correctly and completely.	94.5%	5.4%	0	0	0
		6. Pay attention to referring the patient to answer the consultant physician’s questions and complete the data.	92.7%	7.2%	0	0	0
		7. Describe the patient’s non-verbal signs (facial expressions, etc.) realistically.	90.9%	9.0%	0	0	0
		8. Describe the patient’s economic and cultural conditions and available facilities correctly.	87.2%	10.9%	1.8%	0	0
		9. Send the necessary data to the consultant physician via platforms or virtual spaces.	78.1%	20.0%	0	0	1.8%
	Professional ethics	10. Share the decision made with the consultant physician’s participation with the patient if he or she is conscious.	81.8%	16.3%	1.8%	0	0
		11. Feel responsible for the patient and assigned tasks and gain the trust of the consultant physician.	92.7%	7.2%	0	0	0
		12. Be open to criticism and logic, and show flexibility in the face of opposing opinions of the consultant physician.	92.7%	7.2%	0	0	0
		13. Be aware of the limitations of his knowledge and experience and ask for advice in a timely manner.	90.9%	9.0%	0	0	0
		14. Express it appropriately in cases of error and wrong actions.	85.4%	14.5%	0	0	0
	Awareness and management of emotions	15. Identify stressful situations and manage his stress and emotions to express complete data.	92.7%	7.2%	0	0	0
	Communication skills	16. Communicate with the patient and his family and obtain the necessary data.	78.1%	21.8%	0	0	0
		17. Provide patients and their families with the necessary information and training.	58.1%	40.0%	1.8%	0	0
		18. Recognize the appropriate times to contact the consultant physician and his response.	81.8%	9.0%	3.6%	5.7%	0
		19. Recognize the consultant physician’s response (careful/casual) from the tone of verbal communication.	81.8%	16.3%	0	0	1.8%
		20. Maintain communication with the consultant physician to help resolve the consequences of the decision and follow up on the treatment process.	94.5%	5.4%	0	0	0
	Diagnosis and patient management	21. Recognize teleconsultation emergencies.	92.7%	7.2%	0	0	0
		22. Recognize situations where diagnostic and cognitive errors occur.	81.8%	12.7%	0	0	5.4%
	Collaborative decision-making	23. Select individuals from colleagues and form a team for collaborative decision-making.	54.9%	30.9%	12.7%	0	1.8%
		24. Bracket (temporarily set aside) your pre-made decision and explain the situation fully to the consultant physician.	89.0%	9.0%	0	0	1.8%
		25. Show courage and boldness in expressing your opinions.	70.9%	25.4%	1.8%	0	1.8%
Consultant physician	Receiving and presenting data	26. Listen carefully to the referrnig physician’s presentation.	94.5%	5.4%	0	0	0
		27. Use different methods to evaluate and confirm the data received about the patient (use multiple sources).	96.3%	3.6%	0	0	0
		28. Pay attention to completing the data expressed by the referrnig physician.	96.3%	3.6%	0	0	0
		29. Take notes and summarize oral information.	52.7%	43.6%	3.6%	0	0
	Professional ethics	30. Be responsible for the acute conditions of the physician colleague and allocate sufficient time to provide advice.	94.5%	5.4%	0	0	0
		31. Avoid any gender or ethnic discrimination by requesting timely and accurate advice to patients.	90.9%	9.0%	0	0	0
		32. Do not hesitate to transfer your knowledge and experience and prioritize the patient’s life over receiving financial rewards.	94.5%	3.6%	0	0	1.8%
		33. Pay attention to the use of technology to promote equity in health and reduce socioeconomic gaps in care for all patients.	89.0%	9.0%	0	0	1.8%
	Awareness and management of emotions	34. Help reduce the stress of the referrnig physician during the consultation.	90.9%	7.2%	0	0	1.8%
		35. Answer the questions of the referrnig physician calmly and patiently.	92.7%	7.2%	0	0	0
		36. During the procedures, understand the concerns of the referrnig physician, try to calm him/her down and create a supportive atmosphere for him/her.	94.5%	5.4%	0	0	0
		37. Understand the stressful conditions of the responsible physician of the patient who wants advice.	90.9%	9.0%	0	0	0
		38. Understand the possibility of the referrnig physician’s stress affecting the examination and history taken and the data provided.	92.7%	7.2%	0	0	0
	Communication skills	39. Choose the appropriate communication channel according to the need for effective communication (such as eye contact, speaking speed, tone, body language).	72.7%	27.2%	0	0	0
		40. Explain the decision-making process or procedure and the consequences of the decision to the referrnig physician clearly and completely	98.1%	1.8%	0	0	0
		41. Demonstrate the ability to mentally visualize the referrnig physician’s situation.	76.3%	21.8%	0	0	1.8%
	Collaborative decision-making	42. Consider the possibility of the referrnig physician’s overdiagnosis when hearing his diagnosis.	92.7%	7.2%	0	0	0
		43. Pay attention to the types of differential diagnoses, not just the definitive diagnosis of the referrnig physician.	96.3%	3.6%	0	0	0
		44. Consider the level of literacy and competence of the referrnig physician in decision-making.	90.9%	9.0%	0	0	0
		45. Pay attention to getting feedback from the referrnig physician.	81.8%	16.3%	1.8%	0	0
both physicians	Professional ethics	46. Pay attention to maintaining the privacy and confidentiality of patient information when sending and receiving images to the consultant physician.	98.1%	1.8%	0	0	0
		47. Both physicians should respect each other and after the consultation, the consultant physician should appreciate the consultant physician.	81.8%	18.1%	0	0	0
		48. Pay attention to the patient’s and family’s right to choose and their consent in making decisions and do not make decisions for the patient.	81.8%	16.3%	0	1.8%	0
		49. Share the decision made with the consulting physician’s participation with the patient if he or she is conscious.	83.6%	12.7%	3.6%	0	0
		50. Feel responsible for the acute conditions of the collaborating physician and allocate sufficient time to provide advice.	92.7%	7.2%	0	0	0
		51. Be familiar with the principles of reflection.	60.0%	40.0%	0	0	0
		52. Pay attention to reviewing and reflecting on the decision made for the greater benefit of the patient.	81.8%	18.1%	0	0	0
		53. Pay attention to reflection in consultations to learn more about the disease and acquire skills for subsequent consultations.	83.6%	16.3%	0	0	0
	Teleconsultation and use of technology	54. Be familiar with the advantages and applications of teleconsultation (unfavorable weather, for difficult-to-reach places, in complex decisions, in times of uncertainty in decision-making, lack of awareness of the consequences of the decision, to confirm or reject the subjective diagnosis of the consultant physician, etc.).	74.5%	25.4%	0	0	0
		55. Be familiar with the limitations (lack of direct vision of the patient, conducting the consultation in an inappropriate time and place, etc.) and how to deal with them.	70.9%	29.0%	0	0	0
		56. Pay attention to the use of teleconsultation to help make better decisions.	92.7%	5.4%	1.8%	0	0
		57. Be familiar with the legal responsibilities of the consultant physician and the consultant physician during teleconsultation.	96.3%	3.6%	0	0	0
		58. Establish a suitable environment during video consultations by using appropriate background, lighting, sound, framing, and clothing, while maintaining privacy.	50.9%	47.2%	1.8%	0	0
		59. Use technology for greater interaction with each other.	63.6%	36.3%	0	0	0
	Diagnosis and patient management	60. Understand the differences in the distribution of facilities in different regions and the economic issues of patients, and manage patients in situations with limited facilities.	96.3%	3.6%	0	0	0
		61. Prioritize stabilizing the patient’s physical condition	94.5%	5.4%	0	0	0
		62. Consider patient referral and in-person management when necessary	96.3%	3.6%	0	0	0
		63. Consider reducing the complications of the disease in decision-making.	94.5%	5.4%	0	0	0
	Collaboartive decision-making	64. Pay attention to the role and importance of collaborative clinical reasoning in better decision-making.	94.5%	5.4%	0	0	0
		65. Apply the skill of thinking aloud.	76.3%	23.6%	0	0	0
		66. Pay attention to clear communication about the decision and its reasons.	92.7%	7.2%	0	0	0
		67. Pay attention to creating opportunities for the other physician to speak and express their opinions.	92.7%	7.2%	0	0	0
		68. Be tolerant of ambiguity and actively listen to each other’s conversations.	96.3%	3.6%	0	0	0
		69. Apply ways to increase decisiveness in decision-making (decisive decision-making).	94.5%	5.4%	0	0	0

Table 3, shows the results of the second Delphi round. Three competencies were removed from the list of competencies due to agreement below 70%.

**Table 3:** The percentage of agreements in the second round of Delphi

The role of the physician	Competency domains	Competency	necessary	useful	ineffective	harmful	I cannot give an opinion
Referring physician	Communication skills	1) Provide the patient and his family with the necessary information and training.	85.4%	10.9%	0	0	3.6%
	collaboartive decision-making	2) Select individuals from among colleagues and form a team for collaborative decision-making.	30.9%	60.0%	59.0%	0	0
Consultant physician	Receiving and presenting data	3) Take notes and summarize oral information	49.0%	49.0%	0	0	1.9%
Both physicians	Professional ethics	4) Be familiar with the principles of reflection.	94.5%	5.4%	0	0	0
	teleconsultation and use of technology	5) Create a suitable environment during video consultations by using appropriate background, lighting, sound, framing, and clothing, while maintaining privacy.	41.8%	47.2%	23.6%	0	7.2%
		6) Use technology for greater interaction with each other.	83.6%	16.3%	0	0	0

## Discussion

This study endeavored to determine the expected competencies of specialist physicians involved in teleconsultation. Specialist medical competencies include a combination of knowledge, skills, and attitudes essential for effective patient care [22]. To conduct a teleconsultation, the referring physician is on one side of the consultation process, and the consultant physician is on the other side of the consultation, in this process competencies are required for each physician and both physicians involved. In this study, the main competency domains were extracted as the domains of “receiving and presenting data”, “professional ethics”, “awareness and management of emotions”, “communication skills”, “diagnosis and patient management”, “collaborative decision-making”, and “teleconsultation and use of technology”. In line with the results, Greenhalgh et al. and Pedersen et al. stated that teleconsultation requires specific competencies for both the referring and the consultant physician. For the consultant physician, key competencies include technological skills, communication abilities, triage and management, ethical considerations, and clinical judgment [23, 24].

The domain of “receiving and presenting data” is a pillar of effective teleconsultation, underscoring the critical importance of accurate information transfer in the absence of physical presence. Core competencies in this area include summarizing and classifying key patient data, analyzing information to formulate primary consultation questions, and presenting data completely and accurately. Far from merely administrative, these competencies are foundational to the ACGME frameworks for patient care and medical knowledge. Our analysis suggests that this domain spans multiple CanMEDS roles. These include the medical expert (e.g., knowing which data are critical and formulating pertinent questions) and the collaborator (e.g., effectively communicating findings to a colleague to develop a shared plan) [25, 26]. Supporting this, Lazauskaitė et al. emphasize that reliable data transfer and transparency are essential to overcoming challenges of patient identification [27]. Similarly, Badowski et al. connect these technical competencies to broader clinical context by emphasizing the need to “understand how cultural differences and social background impact telehealth” [28].

“Professional ethics” is one of the vital domains in teleconsultation. This study identifies key ethical competencies— such as patient-centered decision-making, health equity, and privacy— as essential in this domain. While these competencies align with the broad CanMEDS professional role and ACGME frameworks, a more detailed analysis maps them to specific roles: patient-centered decision-making corresponds directly to the medical expert role, addressing health equity is central to the health advocate role, and maintaining privacy represents a fundamental aspect of the professional role. In the ACGME framework, these competencies are primarily integrated into the professionalism and systems-based practice competencies [25, 26]. These findings are consistent with existing literature. For instance, Capello and colleagues highlight patient autonomy, equity, privacy, and the quality of the patient-provider relationship as central ethical concerns [29]. Other studies further emphasize the importance of maintaining the physician-patient relationship, respecting patient dignity, and ensuring informed decision-making [30]. Similarly, Pacheco-Fuentes et al. note that ethical and professional considerations are crucial in teleconsultation [31].

In teleconsultation, communication occurs between two physicians: the referring physician and the consultant. This process requires specific competencies, including effective information exchange, maintaining communication continuity to review decision outcomes, and ensuring timely interactions. These competencies epitomize the CanMEDS communicator role and the ACGME interpersonal and communication skills competency [25, 26]. Supporting this domain, Andrews et al. emphasize the necessity of knowing how to communicate effectively via telephone when a telehealth consultation is required [32]. Similarly, Pacheco-Fuentes et al. assert that “communication skills are paramount in telemedicine” [31].

The competency domain of “awareness and management of emotions” in teleconsultation includes competencies such as identifying personal stress triggers, employing self-regulation strategies to maintain composure, responding with patience and empathy, acknowledging the concerns of the referring colleague, and actively creating a supportive environment. These competencies are directly aligned with the professional role and communicator role of the CanMEDS framework and map to the interpersonal and communication skills and professionalism competencies within the ACGME milestones [25, 26]. Empirical evidence indicates that teleconsultation may predict burnout syndrome [33], highlighting the importance of establishing positive teleconsultation experiences through emotional awareness, stress management, and creating a calm environment [34, 35]. Furthermore, Nguyen et al. emphasize preparatory measures for stress management, including reviewing relevant documents and clinical notes prior to consultations [36].

Furthermore, the domain of “collaborative decision-making” powerfully illustrates several core teleconsultation competencies, such as applying the thinking aloud skill, creating opportunities for the other physician to speak, and active listening. The “thinking aloud” skill, while rooted in clinical reasoning (medical expert), is employed here as a transparent method to share one’s thought process with a colleague, thereby explicitly demonstrating its alignment with the CanMEDS collaborator role and also reflecting the ACGME systems-based practice competency, particularly in working within inter-professional teams to enhance patient safety and care quality [25, 26]. While the significance of inter-physician collaboration in teleconsultation is well-documented across specialties, with studies demonstrating its capacity to modify treatment strategies and improve efficiency, significant implementation gaps persist. These are notably evident in the inadequate adoption of structured templates designed to facilitate this collaborative process [5].

The domain of “diagnosis and patient management” in teleconsultation encompasses essential competencies. These include understanding regional differences in healthcare resources and patients’ economic circumstances to manage cases with limited facilities, prioritizing stabilization of the patient’s physical condition, considering referral and in-person management when necessary, and making decisions aimed at reducing disease-related complications. These competencies are primarily anchored in the CanMEDS medical expert role, while also extending into the leader role through the management of regional resource constraints, and the health advocate role by addressing patients’ economic situations. Within the ACGME framework, this domain is fundamentally tied to both patient care and medical knowledge competencies [25, 26]. While Verma et al. have demonstrated the value of teleconsultations for chronic disease management and triage, persistent concerns regarding diagnostic accuracy and treatment decisions highlight the necessity for resource-aware clinical reasoning in this context [37].

Finally, the competency domain “teleconsultation and use of technology” integrates skills that align with the ACGME core competency frameworks and CanMEDS roles. This area represents competencies including understanding the clinical applications and limitations of teleconsultation, the ability to use technology to enhance decision-making, and adherence to ethical-legal standards. These competencies reflect a combination of multiple roles in these two frameworks: the technical skill of the medical professional, the ethical accountability of the professional, the systems awareness of the leader, the commitment to justice of the health advocate, and the adaptive collaboration of the communicator [25, 26]. Catapan et al. stress that physicians must demonstrate familiarity with various technologies, including telephone systems, email, and videoconferencing solutions [38]. Consistent with this, Sousa et al. confirm that technological competency is critical for the effective delivery of telehealth services [39].

During the Delphi rounds, competencies such as “selecting individuals from among colleagues and forming a team for joint decision-making,” “taking notes and summarizing oral information,” and “creating an appropriate environment during video consultation using suitable background, lighting, sound, framing, and clothing while maintaining privacy” were eliminated. While it can be inferred that these competencies may not be effective given the conditions of consultation in emergencies and remote areas [40, 41], though alternative interpretations are possible. For instance, the panel may have considered these skills as foundational prerequisites for any professional medical interaction, rather than unique competencies specific to teleconsultation. Alternatively, they might have perceived items like “taking notes” as general professional practices that do not require explicit inclusion in a specialized framework.

### Limitations

This study has several limitations. First, the Delphi method relies on expert opinions, which can introduce bias based on the experiences and perspectives of participants. If the selected experts had similar backgrounds or limited experience in teleconsultation, this could have skewed the results. To overcome this limitation, we engaged individuals with diverse experiences and disciplines in the study. Second, the study identified competencies but did not address how training for these competencies is implemented in curricula or how they are assessed in clinical practice. Without clear implementation strategies, the findings may remain theoretical. Third, there is a lack of studies on the competencies of physician-to-physician teleconsultation. Due to the absence of similar research in this area, it was difficult to compare the results with other studies.

### Strengths of the study

This study has several key strengths, including the participation of experienced professionals, which ensures expert insights and reliable findings. In addition, the competency framework creates a shared understanding of the skills, attitudes, and knowledge required to succeed in the role of both physicians involved in teleconsultation. Furthermore, this study provides a first step in identifying learning objectives for educational programs by providing a list of physician-to-physician teleconsultation competencies.

Moreover, the identification of competencies can serve as a basis for the development of competency-based curricula, competency-based instruction, and competency-based assessment. Next, Entrustable Professional Activities (EPAs) and milestones should be developed to design the programmatic assessment.

### Suggestions for future research

For future studies, it would be valuable to investigate the implementation of these competencies in structured physician training programs (clinical simulation and problem-based learning) and evaluate their impact on teleconsultation outcomes. In addition, research should focus on developing validated assessment frameworks, such as competency-based guidelines or real-time peer assessments, to measure proficiency in physician-to-physician teleconsultation.

## Conclusion

With the expansion of digital technologies and the increasing need to access teleconsultation in remote areas or emergencies, physician-to-physician teleconsultation has emerged as an essential tool in the health system. This study suggests that the major competencies for physician-to-physician teleconsultation include the general areas of “receiving and presenting data”, “professional ethics”, “awareness and management of emotions”, “communication skills”, “diagnosis and patient management”, “collaborative decision-making”, and “teleconsultation and use of technology”. While some competency domains, like “professional ethics”, “awareness and management of emotions”, and “communication skills” may appear abstract, they support key aspects of daily clinical practice. These competencies aid in facilitating communication, building trust, and maintaining professionalism during teleconsultations. Additionally, competencies related to “diagnosis and patient management”, “collaborative decision-making”, and “teleconsultation and use of technology” contribute to the practical implementation of teleconsultations. Future research could investigate whether enhancing these skills through targeted training programs better prepares physicians to effectively manage real-world teleconsultation scenarios and, consequently, improves patient care in everyday practice.

Developing and implementing specialized training programs and professional standards based on these competencies could elevate the quality of physician-to-physician teleconsultation and promote equitable access to healthcare services, particularly in underserved and emergency contexts. This study provides a scientific basis for further development of specialized training programs and for enhancing teleconsultation quality.

## Supplementary Information

Supplementary Material 1.

## Data Availability

Data is provided within the manuscript.

## Abbreviations

ICTs: Information and Communication Technologies
CBE: Competency-based education
EPAs: Entrustable Professional Activities
CanMEDS: Canadian Medical Education Directions for Specialists
ACGME: Accreditation Council for Graduate Medical Education
