# Supplementary material for: Essential competencies for an effective physician-to-physician teleconsultation
Source: BMC Med Educ. 2025 Nov 25;26:11. doi: 10.1186/s12909-025-08362-6 (PMC12771948; doi:10.1186/s12909-025-08362-6)
Supplement: Supplementary file 1 — Supplementary Material 1. [file 12909_2025_8362_MOESM1_ESM.docx]

# Appendix 1: INTERVIEW GUIDE

**TITLE:** Essential competencies for an effective physician-to-physician teleconsultation

**Introduction:**
• Begin by introducing yourself to the participants, stating your full name (first and last name).
• Briefly explain the purpose of the study and emphasize that participation is voluntary.
• Confirm the participant’s willingness to proceed and reassure them of their right to withdraw at any time, as well as the confidentiality and privacy protections outlined in the information sheet and consent form.
• Provide an estimate of how long the interview is expected to take.

**Section A:**  Ask the participant to introduce themselves and ask about their work experience and field of expertise.

**Section B:** Guiding questions:

1. Could you recall a recent teleconsultation experience and describe the decision-making process in which you assisted the consulting physician?
2. What factors influence teleconsultation outcomes in your experience?
3. What key considerations do you prioritize during teleconsultations?
4. What competencies should be emphasized when training residents in teleconsultation practices?
5. How do you manage clinical data collection during teleconsultations?
6. What infrastructure requirements facilitate optimal physician-to-physician teleconsultations for improved patient decision-making?
7. For which patient cases do you typically utilize teleconsultation, and how does patient involvement factor into the decision-making process?
8. How do you typically evaluate and follow up on teleconsultation outcomes?
9. Have you encountered specific challenges or limitations in this process, and if so, what mitigation strategies have you employed?

As interviews progressed, questioning was strategically guided toward the study objectives. **Section C:** Probing techniques were systematically employed throughout each interview, including:

- "Could you elaborate further on this?"
- "Would you please expand on that point?"
- "You mentioned [specific concept] - could you clarify what you mean by that?"
